# Effects of Chinese Medicine on the Survival of AIDS Patients Administered Second-Line ART in Rural Areas of China: A Retrospective Cohort Study Based on Real-World Data

**DOI:** 10.1155/2022/5103768

**Published:** 2022-01-27

**Authors:** Yantao Jin, Miao Zhang, Yanmin Ma, Feng Sang, Pengyu Li, Chunling Yang, Dongli Wang, Huijun Guo, Zhibin Liu, Qianlei Xu

**Affiliations:** ^1^Department of Acquired Immune Deficiency Syndrome Treatment and Research Center, First Affiliated Hospital of Henan University of Chinese Medicine, Zhengzhou, China; ^2^Henan Key Laboratory of Viral Diseases Prevention and Treatment of Chinese Medicine, Henan University of Chinese Medicine, Zhengzhou, China; ^3^The First Clinical Medical School, Henan University of Chinese Medicine, Zhengzhou, China; ^4^Center for AIDS/STD Control and Prevention, Center for Disease Control and Prevention of Henan Province, Zhengzhou, China

## Abstract

**Objectives:**

Chinese medicine (CM) improves the symptoms of patients with acquired immune deficiency syndrome (AIDS) and prolongs their survival. This real-world study aimed to evaluate the effects of CM on the survival of AIDS patients administered second-line antiretroviral therapy (ART).

**Methods:**

We conducted a retrospective cohort study of the medical records of patients with AIDS who switched to second-line ART between January 2009 and December 2014. Patients were divided into ART and CM + ART groups. Propensity score matching (PSM) was performed to correct for biases between groups. Kaplan–Meier analysis and the log-rank test were used to compare survival rates, and Cox regression models were employed to identify factors significantly associated with survival.

**Results:**

The study population (*n* = 4180) was comprised of the CM + ART group (*n* = 855) and the ART group (*n* = 3325). After 1 : 2 PSM, 855 patients in the CM + ART group and 1699 in the ART group were selected for analysis. Patients in the CM + ART group were followed for 4246.8 person-years, and the mortality rate was 2.12/100 person-years. Patients in the ART group were followed for 8381.2 person-years, and the mortality rate was 2.91/100 person-years. Cox regression model analysis revealed that patients in the CM + ART group survived significantly longer than those in the ART group (hazard ratio: 0.73 and 95% confidence interval: 0.57–0.93). Gender, age, symptoms, CD4 cell counts, and viral loads were independently associated with the survival of AIDS patients treated with second-line ART.

**Conclusions:**

CM significantly improved the survival rate of AIDS patients treated with second-line ART.

## 1. Introduction

The survival of patients with acquired immune deficiency syndrome (AIDS) improved after the introduction of antiretroviral therapy (ART) over 30 years ago. However, the global prevalence of AIDS (37.7 million cases in 2020) remains a serious threat to public health [1]. Although ART is a well-established and effective therapy for AIDS, the increasingly common problems of duration of ART, nonadherence, drug resistance, and treatment failure represent challenges to achieving better responses to treatment [2, 3]. In 1997, a second-line ART regimen, including one protease inhibitor (PI) and two nucleoside reverse transcriptase inhibitors, was recommended as the preferred treatment strategy for patients with AIDS [4].

An increasing number of patients with AIDS have been administered this second-line regimen with the expectation of a significant benefit. For example, a study on patients with AIDS in Asia found that 19% of patients were administered second-line ART [5]. However, some patients failed to respond to second-line ART [6–9]. The WHO recently recommended third-line ART for patients with AIDS. Unfortunately, access to this treatment is restricted by its high cost and barriers to its implementation [3]. Thus, maximizing the efficacy and access to first- or second-line regimens is an emerging global priority. We believe that Chinese medicine (CM) may serve this purpose for the reasons outlined below.

CM has been used for thousands of years as an effective treatment for acute viral infections; recent examples are COVID-19, caused by SARS-CoV-2 [10], influenza caused by influenza A virus subtype H1N1 [11], as well as severe acute respiratory syndrome [12]. Moreover, CM plays an important role in the treatment of AIDS, particularly in China. Previous studies found that CM based on first-line ART could ameliorate symptoms and signs of AIDS [13], improve quality of life [14], increase CD4+ T cell counts [15], promote adaptive immunity, and prolong survival [16]. Increasing numbers of patients with AIDS in China are taking CM along with second-line ART, but its effects are unclear. Therefore, we conducted a retrospective cohort study using standard medical records to evaluate the effects of CM on the survival of AIDS patients administered second-line ART.

## 2. Methods

### 2.1. Study Setting

During the 1990s in Henan Province, which is located in the center of China, patients with AIDS were infected with commercially available blood as well as with illegally collected blood plasma [17]. From 2003, ART was administered to these patients in accordance with the guidelines of the National Free Antiretroviral Treatment Program (NFATP), and second-line ART, which comprises lamivudine (3TC) + tenofovir (TDF) + lopinavir/ritonavir (Lpv/r), was introduced in 2009 in Henan Province [18]. Since 2004, the State Administration of Traditional Chinese Medicine has sponsored a national CM–AIDS Treatment Trial Program (NCMATP) for patients with AIDS. All of the study patients voluntarily chose to participate in the NCMATP and provided signed informed consent. The patients in the NCMATP of Henan were given the patented Chinese drug yi ai kang (containing substances such as ginseng, Huangqi, Chaobaishu, Tuckahoe, Chinese angelica, Chuanxiong, Baishao, and Scutellariae) free of charge (five capsules three times a day) and were required to record medical information related to their AIDS condition on a monthly basis [19].

### 2.2. Study Design and Patient Population

We conducted a retrospective cohort study of the standard medical records of patients with AIDS who were administered second-line ART in rural areas of Henan Province, where such patients were enrolled in NCMATP before 2009. Patients who switched to second-line ART from January 2009 to December 2014, who were older than 18 years or younger than 65 years, were included. Individuals with incomplete baseline data were excluded from the study. The start of the analysis period was defined as the time patients switched to second-line ART. Survival was defined as time to death on December 30, 2019, or 6 years after treatment commenced. Patients' data were censored if their dates of death were not recorded, if they were lost to follow-up, if they withdrew from treatment with CM plus or ART, or if they were alive when the study was completed.

### 2.3. Data Collection and Patients' Characteristics

Patients' clinical and demographic information was acquired from the standard medical records of the NCMATP or NFATP, Henan. This information included age; gender; marital status; ethnicity; education; occupation; route of infection; year of confirmed human immunodeficiency virus (HIV) infection; dates when ART and second-line ART commenced; administration of CM; symptoms of AIDS such as skin lesion, thrush, oral hairy leukoplakia, persistent diarrhea (>1 month), constant fever (>38°C, >1 month), severe bacterial infections, pulmonary tuberculosis, chronic herpes simplex infection, herpes zoster; CD4 cell count; viral load (VL); date of death; and date censored. If patients had one type of symptom, this variable was recorded as “yes.” CD4 cell counts and VLs were those recorded within 6 months from the commencement of second-line ART, and if no value was entered during this interval, the variable was defined as missing. Patients were divided into ART and CM + ART groups according to their enrollment in the NCMATP.

### 2.4. Ethics Approval

This study was approved by the institutional review board of the first hospital affiliated to Henan University of Traditional Chinese Medicine (2019HL-068). Individual informed consent was not obtained because this analysis used currently existing data collected during the course of routine treatment and the data were reported in aggregate without the use of individual identifying information.

### 2.5. Data Analysis

Demographic and clinical data before and after matching were compared between patients in the CM + ART and ART groups. Categorical variables are expressed as numbers with percentages and were compared using the Fisher exact or chi-square tests. The study of data resources from real-world data (RWD) is defined as a real-world study (RWS) [20]. Propensity score matching (PSM) is widely used to more effectively control for baseline imbalances among groups in RWS [21]. Propensity scores were generated using a multivariable logistic regression model with the group as the dependent variable. Baseline variables such as age, gender, marital status, education, route of infection, time on HIV positive, time on ART before second-line, symptoms, CD4 cell counts, and VL were independent variables. Matching of the CM + ART and ART groups in 1 : 2 ratios was performed using the nearest neighbor method with a fixed caliper width = 0.1. After matching, the standardized difference (SD) was used to assess the degree of balance among baseline variables. SD ≤ 0.1 indicates a high degree of balance [22].

The survival rates of patients in the CM + ART and ART groups were calculated using the Kaplan–Meier method and compared using the log-rank test. Univariate and multivariable Cox proportional hazards regression models of the matched data were performed to identify factors that influenced the survival of patients with AIDS. The results of the Cox proportional hazards regression model are presented as the hazard ratio (HR) and 95% confidence interval (CI).

The PSM method was performed using the “MatchIt” package, SD was calculated using the “stddiff” package, and the Cox proportional hazards regression model is included in the “Survival” package in R 3.6.2 software. Significant differences are defined as *P* < 0.05.

## 3. Results

### 3.1. Subjects

We included 4180 patients with AIDS treated with second-line ART in rural China from 2009 to 2014. Patients were allocated into the CM + ART (*n* = 855) and ART (*n* = 3325) groups. The results before and after performing 1 : 2 PSM of 855 patients in the CM + ART group and 1699 patients in the ART group are summarized in Table 1. There were significant differences (*P* < 0.05) in age, route of infection, time on ART before second-line, CD4 cell counts between the CM + ART and ART groups before matching, and there were no significant differences after PSM.

**Figure 1:**
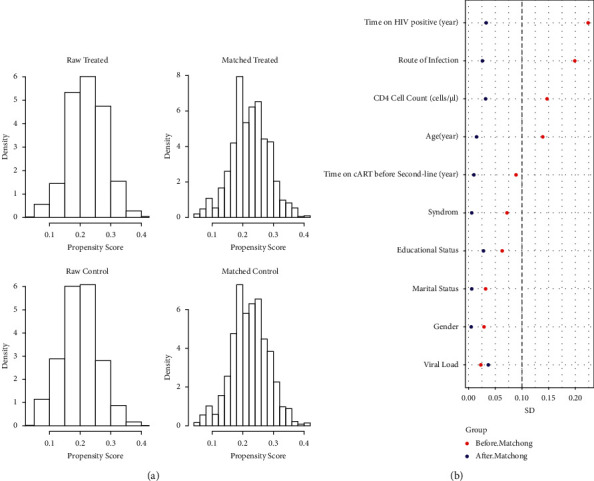
The balance between the CM + ART and ART groups after PSM. (a) Histograms of the propensity scores of the two groups before and after matching; (b) standardized difference (SD) of baseline characteristics of the two groups before and after matching.

To further assess balance, we analyzed the histograms of the propensity score distribution and SD of variables in the two groups before and after PSM.  Figure 1(a) presents a histogram showing the balanced distribution of propensity scores in the two groups according to PSM.  Figure 1(b) presents a scatter diagram showing that the SD of all variables after PSM was <0.1.

### 3.2. Survival Analysis of Patients with AIDS Administered Second-Line ART

**Figure 2:**
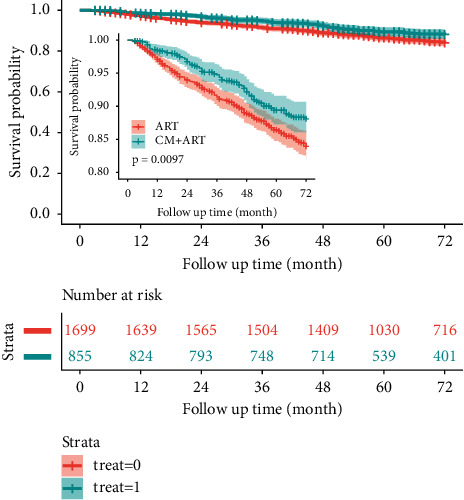
Kaplan–Meier survival analysis of patients with AIDS treated with second-line ART after PSM.

Among the 2544 patients with AIDS after PSM who were followed for 12,628 person-years, 334 died, and the mortality rate was 2.64/100 person-years. Among the 855 patients with AIDS in the CM + ART group who were followed for 4246.8 person-years, 90 (6.5%) died, and the mortality rate was 2.12/100 person-years. Among the 1699 AIDS patients in the ART group who were followed for 8381.2 person-years, 244 (6.5%) died, and the mortality rate was 2.91/100 person-years. The survival curves of the CM + ART and ART groups are shown in Figure 2. The HR for mortality of patients in the CM + ART group compared with those in the ART group was 0.73 (95% CI: 0.57–0.93).

### 3.3. Factors Associated with the Survival of Patients with AIDS Treated with Second-Line ART

**Figure 3:**
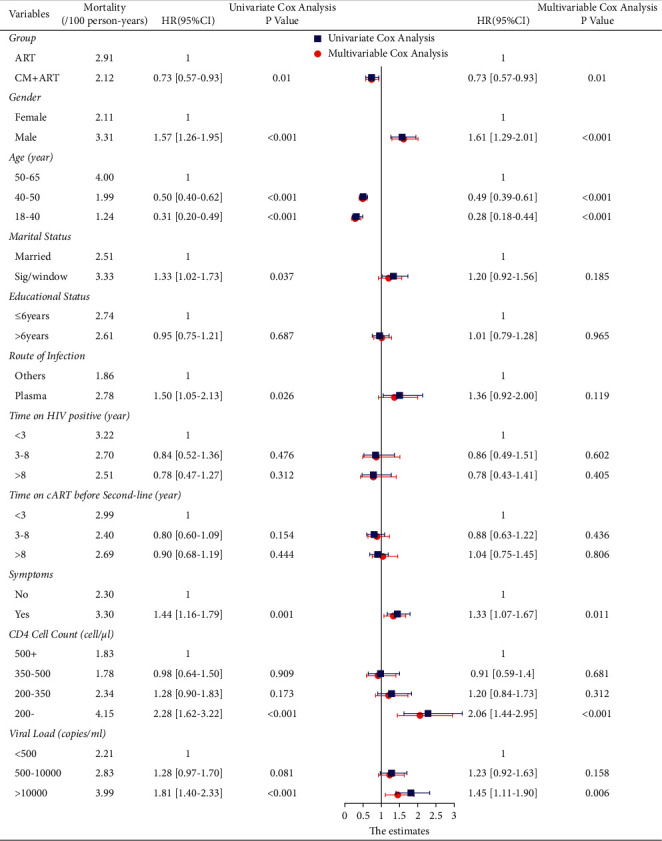
The Cox proportional hazards model analysis showing the significance of the associations of variables with mortality of patients with AIDS.

CM, gender, age, symptoms, CD4 cell count, and VL were independently associated with mortality of patients with AIDS administered second-line ART. The results of the multivariable model showed that the HRs of patients aged 40 years to 50 years and 18 years to 40 years were 0.49 (95% CI: 0.39–0.61) and 0.28 (95% CI: 0.18–0.44), respectively, compared with patients aged 50 to 65 years. The HR of patients with <200 CD4 cells/*µ*l was 2.06 (95% CI: 1.44–2.95). The details of the results of the analyses using the univariate and multivariable Cox proportional hazards models are presented in Figure 3.

## 4. Discussion

Here we evaluated the efficacy of CM administered to AIDS patients treated with second-line ART. For this purpose, we retrospectively analyzed standard medical records of the NCMATP or NFATP for Henan Province based on RWS. There are many advantages of a RWS, such as the validity of the data. However, intrinsic selection biases are associated with the data resource, which influenced the design of the present study. PSM was used to control for baseline imbalances among groups in the study. After we conducted PSM here, the selection biases were well controlled as indicated by SDs <0.1 for all variables.

We show here that the overall mortality rate of patients with AIDS administered second-line ART was 2.64/100 person-years, which is lower than that reported by most studies on the mortality rates of patients with AIDS after initial or no second-line administration of ART [17, 23, 24]. The data are as follows: 8.53 person-years in Guizhou [17], 5.1/100 person-years in Sichuan [23], and 3.9/100 person-years in Henan [24]. These data suggest that second-line ART is more effective for reducing mortality rates. Another study conducted in Africa and Asia in 2010 reported a mortality rate equal to 4.42/100 person-years after patients switched to second-line ART [25]. According to the Global HIV Statistics Fact Sheet published by the WHO, the mortality rate of AIDS patients sharply declined [1]. The differences among the results of these studies may be explained by differences in patients' characteristics, the numbers of patients, treatment strategies, length of study, and study date.

In the present study, the mortality rate of patients with AIDS in the CM + ART group was 2.12/100 person-years and the HR was 0.73, which is comparable with mortality data for the ART group (2.91/100 person-years). These results suggest that CM combined with second-line ART significantly lengthened the survival of patients with AIDS. The theory of CM states that the body is recognized and treated as an entire entity, and diseases are identified as evil spirits that enter the body and cause internal imbalances. The yi ai kang, which was used in the CM + ART group, was taken to invigorate the spleen and supplement qi, nourishing Yin and blood, dispelling wind and clearing heat as the treatment principle, and to achieve the effect of strengthening the root, that is, to increase the body's *positive qi* to improve immune function [26], enhance immune function, and reduce AIDS-associated symptoms [27].

Except for CM, we show here that gender, age, symptoms, CD4 cell count, and VL were significantly associated with the survival of patients with AIDS administered second-line ART. Male patients had a higher risk of death compared with female patients, which is consistent with many studies [17, 28, 29]. Older patients are at higher risk of death because of more comorbidity and may be significantly associated with the failure of second-line ART [30]. Lower CD4 cell counts or higher VLs upon the switch to second-line ART were independent risk factors for time to death, which is consistent with many studies. Patients with a lower CD4 cell count were found to be significantly associated with the failure of second-line ART and have a higher probability of developing different opportunistic infections [8], all of which are more apt to cause death.

The aims of this study were to evaluate the effects of CM and the role of the NCMAPT, specifically, on the survival of AIDS patients taking second-line ART. The curative effects of participation in the project reflect not only the effects of the ART or CM drugs, but likely include humanistic factors as well, such as the provision of more health care, which may have enhanced the positive results of the study. Although we used PSM to control for selection bias, a retrospective cohort study is intrinsically biased. Furthermore, specific important variables associated with mortality, such as adherence to ART, body mass index, anemia, hyperlipemia, and liver injury [31–33], were not recorded when patients switched to second-line ART.

## 5. Conclusions

Our study indicates that patients who were with AIDS administered CM therapy and second-line ART experienced lower mortality compared with patients administered second-line ART alone. CM may therefore enhance the benefit of second-line ART. Prospective studies on the use of pure CM must be performed to confirm our results.

## Figures and Tables

**Table 1:** Comparisons of baseline data of the CM + ART and ART groups before and after PSM.

Variables	Before PSM			After PSM		
	ART (*n* = 3,325)	CM + ART (*n* = 855)	*P* value	ART (*n* = 1,699)	CM + ART (*n* = 855)	*P* value
*Gender*			0.476			0.945
Female	1545 (46.5%)	385 (45.0%)		930 (54.7%)	470 (55.0%)	
Male	1780 (53.5%)	470 (55.0%)		769 (45.3%)	385 (45.0%)	

*Age (years)*			0.002			0.936
18–40	591 (17.8%)	110 (12.9%)		210 (12.4%)	110 (12.9%)	
40–50	1547 (46.5%)	412 (48.2%)		823 (48.4%)	412 (48.2%)	
50–65	1187 (35.7%)	333 (38.9%)		666 (39.2%)	333 (38.9%)	

*Marital status*			0.430			0.930
Married	2728 (82.0%)	712 (83.3%)		1411 (83.0%)	712 (83.3%)	
Single/widow	597 (18.0%)	143 (16.7%)		288 (17.0%)	143 (16.7%)	

*Ethnicity*			0.774			0.740
Han	3311 (99.6%)	851 (99.5%)		1693 (99.6%)	851 (99.5%)	
Others	14 (0.42%)	4 (0.47%)		6 (0.35%)	4 (0.47%)	

*Occupation*			0.712			0.144
Farmer	3209 (96.5%)	828 (96.8%)		1663 (97.9%)	828 (96.8%)	
Others	116 (3.49%)	27 (3.16%)		36 (2.12%)	27 (3.16%)	

*Educational status*			0.112			0.539
≤6 years	1021 (30.7%)	238 (27.8%)		452 (26.6%)	238 (27.8%)	
>6 years	2304 (69.3%)	617 (72.2%)		1247 (73.4%)	617 (72.2%)	

*Route of infection*			<0.001			0.577
Others	719 (21.6%)	120 (14.0%)		1445 (85.1%)	735 (86.0%)	
Plasma	2606 (78.4%)	735 (86.0%)		254 (14.9%)	120 (14.0%)	

*Time on HIV positive (years)*			0.066			0.735
<3	683 (20.5%)	159 (18.6%)		82 (4.83%)	36 (4.21%)	
3–8	1013 (30.5%)	295 (34.5%)		888 (52.3%)	456 (53.3%)	
>8	1629 (49.0%)	401 (46.9%)		729 (42.9%)	363 (42.5%)	

*Time on ART before second-line (years)*			<0.001			0.974
<3	323 (9.71%)	36 (4.21%)		322 (19.0%)	159 (18.6%)	
3–6	1756 (52.8%)	456 (53.3%)		586 (34.5%)	295 (34.5%)	
>6	1246 (37.5%)	363 (42.5%)		791 (46.6%)	401 (46.9%)	

*Symptoms*			0.069			0.924
No	2083 (62.6%)	565 (66.1%)		1118 (65.8%)	565 (66.1%)	
Yes	1242 (37.4%)	290 (33.9%)		581 (34.2%)	290 (33.9%)	

*CD4 cell count (cells/μl)*			0.002			0.902
>500	719 (21.6%)	149 (17.4%)		315 (18.5%)	149 (17.4%)	
350–500	686 (20.6%)	162 (18.9%)		311 (18.3%)	162 (18.9%)	
200–350	973 (29.3%)	301 (35.2%)		588 (34.6%)	301 (35.2%)	
<200	947 (28.5%)	243 (28.4%)		485 (28.5%)	243 (28.4%)	

*Viral load*			0.833			0.678
<500	2069 (62.2%)	536 (62.7%)		1075 (63.3%)	536 (62.7%)	
500–10000	636 (19.1%)	156 (18.2%)		323 (19.0%)	156 (18.2%)	
>10000	620 (18.6%)	163 (19.1%)		301 (17.7%)	163 (19.1%)	

PSM, propensity score matching; HIV, human immunodeficiency virus; ART, antiretroviral therapy.

## Data Availability

The data used to support the findings of this study are available from the corresponding author upon request.
